# Anti-MUC1 Antibody in Nipple Aspirate Fluids Correlates with Tumor Aggressiveness in Breast Cancer: A Feasibility Study

**DOI:** 10.1155/2015/179689

**Published:** 2015-11-29

**Authors:** Ebru Menekse, John McKolanis, Olivera J. Finn, Priscilla F. McAuliffe, Ronald Johnson, Atilla Soran

**Affiliations:** ^1^Division of Surgical Oncology, Department of Surgery, University of Pittsburgh School of Medicine, Magee-Womens Hospital of UPMC, Pittsburgh, PA 15213, USA; ^2^Department of Immunology, University of Pittsburgh School of Medicine, Pittsburgh, PA 15213, USA

## Abstract

Antibodies against MUC1 are found in circulation of breast cancer (BC) patients. We hypothesized that anti-MUC1 antibodies might be present in even a higher concentration in nipple aspirate fluid (NAF) and could be used to predict aggressiveness of BC. Serum and NAF samples were collected from high risk lesions, BC, and healthy contralateral breasts. ELISA was used to measure the amount of IgG, IgM, and IgA against a tumor-specific MUC1 peptide derived from the extracellular tandem repeat domain of MUC1. Tumor characteristics were recorded prospectively; 120 NAF samples were obtained from a total of 77 women in the study. There was no significant difference of anti-MUC1 antibody levels compared to BC with other lesions. Anti-MUC1 IgG level in NAF was higher in triple negative tumors (*P* = 0.02); serum anti-MUC1 IgG levels were significantly higher in patients with ER (−) tumor and recurrent disease (*P* = 0.01); NAF anti-MUC1 IgA levels were significantly higher in patients with LVI and Her2-neu (+) tumors (*P* < 0.05). These results show that NAF could be a reliable biomarker to predict tumor aggressiveness in BC. A larger study will be needed to confirm these data and to investigate the potential of anti-MUC1 antibodies in NAF and serum to predict disease outcome.

## 1. Introduction

Breast cancer (BC) is the most common cancer and it is also the second most frequent cause of cancer deaths among women in the USA, accounting for an estimated 231,840 new cases and 40,290 deaths for women in the USA in 2015 [1, 2]. Improvements in BC survival have come with early diagnosis of high risk women using mammograms and improved chemo- and hormonotherapy regimens for the last 3 decades [3]. However, there is a critical need to develop technically feasible, less invasive, inexpensive, and reliable methods to allow earlier diagnosis, identify aggressive phenotypes, and monitor changes during therapies for better prognosis.

MUC1 is a large o-glycosylated transmembrane protein that is expressed on the apical surface of healthy breast ductal epithelial cells and on other epithelial surfaces such as the gastrointestinal tract, respiratory tract female reproductive tract, and pancreas [4, 5]. Under normal conditions, MUC1 protects the apical cell surface against microbial infection and dehydration [5]. Both premalignant and malignant epithelial cells and nonmalignant but inflamed epithelial cells overexpress abnormal MUC1 [6, 7]. Compared to the normal molecule, abnormal MUC1 is underglycosylated, and the sugars are truncated resulting in exposure of new protein antigenic epitopes. The short sugar epitopes may also stimulate an immune response [8, 9] against antigenically recognizable peptide and glycopeptides epitopes [10]. A potential diagnostic and prognostic value of anti-MUC1 antibodies in sera [11, 12] was studied in the literature but this was not investigated in nipple aspirate fluid (NAF) previously. In contrast to a breast biopsy, aspiration of NAF is a noninvasive method to obtain intraductal material [13]. NAF is composed predominantly of proteinaceous secretions from ductal cells. Therefore, NAF may be representative of the dynamic secretory process of the breast and may include potential markers of carcinogenesis [13–15]. Examples of tumor antigens and oncoproteins that have previously been examined in NAF include prostatic-specific antigen, carcinoembryonic antigen, and c-erb B2 [15–18].

We hypothesized that antibodies against abnormal MUC1 on BC would be present in NAF in higher concentrations in women with BC compared to those with nonmalignant lesions and additionally might be used to predict aggressiveness of BC.

The aim of this feasibility study was to compare anti-MUC1 antibody levels as a diagnostic marker in NAF and in serum of patients with BC and premalignant lesions and in normal breasts. The second objective of this study was to investigate relationship between anti-MUC1 antibody levels in NAF as a predictive marker for tumor aggressiveness.

## 2. Patients and Methods

### 2.1. Study Design

Women enrolled in this study were patients intending to have surgery for treatment of BC, premalignant breast disease, and benign breast disease who had been referred to Breast Surgery Unit of Magee-Womens Hospital of University of Pittsburgh Medical Center. Demographics, pretreatment serum, and NAF samples were collected prospectively after approval of the Institutional Review Board. Exclusion criteria were as follows: previous history of cancer, prior breast surgery, radiation, chemotherapy, or endocrine therapy, current use of hormone replacement therapy (estrogen or an estrogen/progesterone combination), current pregnancy, and lactation. All subjects underwent bilateral breast physical exam and had a mammogram and/or ultrasound and breast MRI if indicated.

Collection of NAF samples was performed on anesthetized patients in the operating room prior to surgery for newly diagnosed BC, high risk lesions, and normal breast. Normal breast was defined as contralateral healthy breast of patients with unilateral breast malignancy or premalignancy and breasts of patients who underwent surgery for cancer risk reduction or for nonproliferative breast lesions.

Patients and tumor characteristics, such as age, size of the tumor (pT), lymph node status (pN), estrogen receptor (ER), progesterone receptor (PR), Her2-neu receptor, nuclear grade, lymphovascular invasion (LVI), extracapsular invasion (ECE), and duration of follow-up, date of recurrence, or death, were collected prospectively.

Overall survival (OS) was calculated from the date of diagnosis to the date of death, and disease free survival (DFS) was measured from the date of diagnosis to the date of local recurrence or distant metastasis.

### 2.2. NAF Collection Procedure

Each breast was massaged for at least 3–5 minutes and the nipple was cleaned with EKG gel and an alcohol wipe. The nipple aspirator (*First-Cyte; Cytyc Health*) was put in place and negative pressure was applied to both breasts at the same time. NAF was collected using 10 *μ*L pipettes and transferred quickly to the laboratory. Depending on the volume of NAF collected, it was stored at −70°C either neat or diluted up to 20-fold with 1x phosphate-buffered saline (PBS).

### 2.3. Detection of Anti-MUC1 Antibody by ELISA

Enzyme-linked immunosorbent assay (ELISA) method was used to detect anti-MUC1 IgG, IgM, and IgA. Antibodies were measured against a synthetic 100-mer MUC1 peptide corresponding to five tandem repeats of the MUC1 polypeptide core tandem repeat region [19, 20] corresponding to the most abnormal, unglycosylated tumor form of MUC1. Briefly, MUC1-coated Immulon wells (*Dynax, Chantilly, VA*) and peptide-negative plates were incubated overnight and washed three times with PBS before addition of 100 *μ*L of 2.5% bovine serum albumin in PBS. Serially diluted plasma (1 : 40 to 1 : 80 in PBS) was added to MUC1-coated plates and incubated at room temperature. Plates were washed 5 times with 100 *μ*L PBS and 0.1% Tween 20 detergent. Alkaline phosphatase-labeled goat anti-human polyvalent IgM, IgG, and IgA (50 *μ*L) (*Sigma-Aldrich, St. Louis, MO*) diluted 1 : 1,000 were added before plates were washed again 5 times with PBS-Tween. Alkaline phosphatase substrate pNPP (100 *μ*L) (*Sigma-Aldrich*) was added. Plates were incubated before the stop solution (0.5 mol/L NaOH) was added. We used the MRX Revelation plate reader (*Thermo Labsystems, Chantilly, VA*) to read absorbance values at 405 nm, which were subtracted from absorbance values obtained from antigen-negative plates to account for nonspecific binding. The color change read as an OD value and that value was directly proportional to the amount of antibody present in NAF and serum. The quantity of anti-MUC1 antibodies (IgG, IgM, and IgA) in the serum was represented as absorbance at OD 405 nm.

### 2.4. Statistical Analyses

All data are presented as mean ± standard deviation. Associations between tumor characteristics and immune-biomarkers level in NAF or serum were examined using the ANOVA test. The difference in levels of NAF and serum MUC1 antibodies between BC, high risk lesions, and normal breast tissue was examined by analysis of ANOVA followed by Tukey's comparison procedure. OS and DFS were measured using Kaplan-Meier method. A *P* value less than 0.05 was considered statistically significant. All statistical tests were performed using SPSS, version 21.0, software (SPSS Inc., Chicago, IL).

## 3. Results

One hundred and twenty NAF samples were collected prospectively from 77 women who underwent breast surgery. Among them there were 52 BC patients but NAF was obtained in 44 breasts with cancer. The rest of 76 NAF samples were collected from the following: 7 (6%) had in situ lesions, 18 (15%) had atypia (15 ductal type, 3 lobular type), and 51 (43%) had normal breast tissue.

The majority of women (93.6%) were older than 40 years. The mean age of women with BC was 54 ± 10 years (range 40–84). The mean follow-up period was 73 ± 18 months with range of 51 to 187 months. There were 4 recurrences, 2 of which were local recurrence and 2 of which were distant metastases. The 6-year OS and DFS were 98.1% and 92.3%, respectively. Tumor characteristics of patients with malignant disease are shown in Table 1.

**Figure 1:**
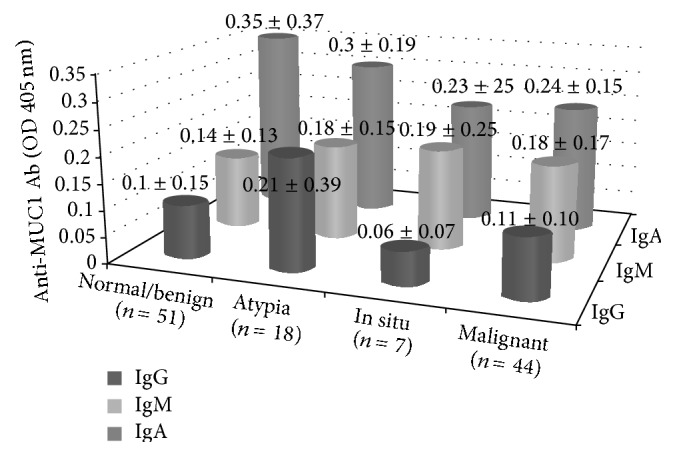
Levels of NAF anti-MUC1 antibodies in patients with different breast lesion (*P* > 0.05). NAF: nipple aspirate fluid.

**Figure 2:**
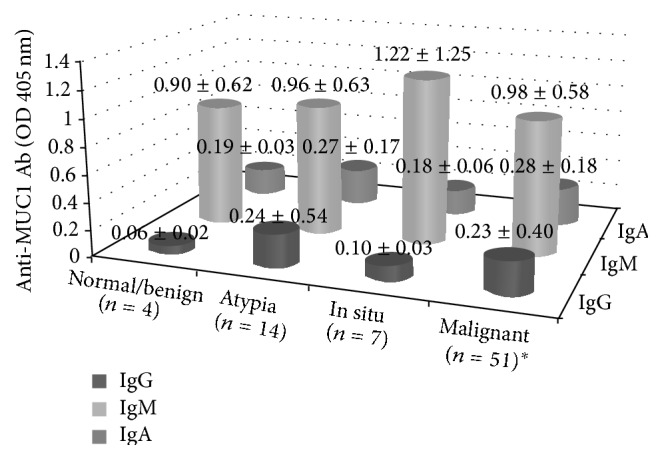
Levels of serum anti-MUC1 antibodies in patients with different breast lesion (*P* > 0.05). ^*∗*^One missing data for serum samples from 52 patients with malignant breast lesions.

There was no significant difference in levels of anti-MUC1 antibodies between BC, in situ lesions, atypical hyperplasia, and normal breast tissue either in NAF or in serum (Figures 1 and 2). However, in the NAF of women diagnosed with BC, anti-MUC1 IgG was higher in triple negative patients (*P* = 0.02). NAF anti-MUC1 IgA levels in cancers with LVI and in Her2-neu positive cancers were significantly higher than those without these features (*P* = 0.005, *P* = 0.02, resp.). High levels of serum anti-MUC1 IgG were significantly associated with ER negative disease and with disease recurrence (both at *P* = 0.01). Serum anti-MUC1 IgM levels were higher in patients who had tumor that was equal or smaller than 2 cm (*P* = 0.03). Statistically significant relationships between NAF or serum levels of anti-MUC1 antibodies as immune biomarkers and tumor characteristics are shown in Table 2. There was no relationship between lymph node positivity, grade, and Ki 67 status and anti-MUC1 antibody levels.

## 4. Discussion

The relationship between MUC1 and tumor characteristics in BC was evaluated in several IHC studies but results of these studies are conflicting. Some studies show that overexpression of MUC1 is associated with better tumor behavior [21, 22], absence of regional recurrence, and distant metastasis, but others demonstrate the opposite [23, 24]. On the other hand, the presence and significance of anti-MUC1 antibodies have not been previously evaluated in NAF.

Measurement of anti-MUC1 antibody levels is more practical and advantageous than determination of MUC1 antigens, because regardless of tumor size, a tiny bit of antigen made by a few premalignant or malignant cells can drain to the nearest lymph node and induce an immune response that can be detected in NAF. Furthermore, according to an interesting study presented by Larrain et al., after serums of breast cancer patients that were combined with incubated breast tissues had different pathologic diagnosis such as normal, benign, and malignant, they found the reduction in anti-MUC1 antibody and antigen reactivity to be more in breast cancer tissues than benign breast tissues [25]. On the other hand, NAF is an inexpensive and feasible method that may reflect the tumor microenvironment. In addition, NAF which is noninvasive method can be easily collected in outpatient department from women, despite the fact that it was collected under general anesthesia from patients who had pathologic diagnosis of breast tumor in our study [15, 18]. Moreover, antibody measurement in NAF enables more correct determination than in serum of anti-MUC1 IgA, because IgA is epithelial surface antibody. In this study, we demonstrated that anti-MUC1 IgG, IgM, and IgA antibodies could be detected in NAF using ELISA and that high level of anti-MUC1 IgG antibody in NAF was significantly associated with triple negative tumor phenotype (ER−, PR−, and Her 2−) and anti-MUC1 IgA antibodies were also significantly higher in the NAF of patients with LVI and Her2-neu-positive disease. These results suggest that high levels of anti-MUC1 antibody in NAF were associated with poor tumor characteristics. However, several immunohistochemical (IHC) studies have also demonstrated that MUC1 expression is associated with ER and PR positivity [21, 22, 24]. According to Rakha et al. high expression of MUC1 was associated with ER positivity, smaller size, and low-grade tumors [22]. They also found a negative association between expression of MUC1 and presence of lymph node metastasis, regional recurrence, and distant metastasis [22]. Garbar et al. showed that higher IHC staining of MUC1 was observed in PR positive tumors [24]. On the other hand, they also showed that MUC1 expression was higher in lymph node metastases when compared to primary tumor tissue [24]. McGuckin et al. showed that high level of MUC1 expression was associated with both axillary node metastasis and ER positivity [23]. Also, in our study, high serum levels of anti-MUC1 antibodies are associated with both ER negative and smaller size tumors. Favorable prognostic effects of MUC1 are seen in association with ER positivity, while poor prognostic may be associated with the inhibition of integrin and E-cadherin mediated cell adhesion and the binding of MUC1 to ICAM-1, which is thought to play a role in cell migration [7, 11, 21]. In addition, high level of anti-MUC1 antibody suggests the immunogenic potential of MUC1 [26]. The literature suggests that MUC1 affords protection against disease progression as a consequence of natural humoral immunity [26], consistent with a significant association of lymph node negativity and improved OS in patients with high level of serum anti-MUC1 IgG and IgM antibody [11, 27]. Studies by von Mensdorff-Pouilly et al. describe an inverse correlation between serum anti-MUC1 antibody and disease extent [27]. Herein, we demonstrated an association between anti-MUC1 IgG in the serum and recurrent disease; in spite of the fact that there was no relationship of lymph node positivity, tumor grade and Ki67 status between anti-MUC1 antibodies levels, there was a statistically significant association between anti-MUC1 IgA in NAF with LVI. Some of these results in the literature seem contradictory in our results. However, these studies have limited the number of samples similar to our study. Therefore, current knowledge about the response of anti-MUC1 antibody of patients with different patterns of tumor receptors is not sufficient, but it was shown that MUC1 expression is related to ER status [23]. It is possible that anti-MUC1 antibody in ER negative tumor does not afford the same protective effect in patients with ER positive BC [28]. The conflicting results belong to recurrence and other tumor characteristics for each of MUC1 and anti-MUC1 antibody may depend on this theory.

In this study, we investigated whether anti-MUC1 antibody, in either NAF or serum, could discriminate between BC, premalignant breast lesion, and normal breast, but we did not demonstrate a significant difference. Ghosh et al. showed using IHC that overexpression of underglycosylated MUC1 localized markedly in normal breast tissue adjacent to tumors [29]. On the other hand, Tang et al. demonstrated that anti-MUC1 IgG immune complex in the serum of patients with BC and benign breast lesion was higher than in healthy women [30]. In our study, there were very few patients that had only benign breast lesions and few healthy women. This limits our demonstration of such findings in serum, because high serum level of anti-MUC1 antibodies can be found in patients with benign breast lesions, after breast inflammation and benign proliferative condition such as pregnancy [6, 31]. In our study discrimination features of anti-MUC1 antibodies were investigated for benign lesions in NAF as well, but it had no significant difference between benign breast lesions and healthy women. A small number of patients with bilateral normal breasts (*n* = 4) and additionally NAF were obtained from contralateral side of premalignant or malignant breasts which might not be considered as benign lesions and normal breasts. Although it is possible that NAF obtained from each breast in the same patient reflects the microenvironment itself. Kuerer et al. reported that Her2-neu in NAF was seen in bilateral breasts from women who had unilateral invasive cancer. They found that levels of Her2-neu were significantly higher in the affected breast than in the unaffected breast [16].

Although our study had a follow-up period of an average of 73 months, we were unable to investigate the relationship between prognosis in BC and immune biomarkers, due to the small sample size of the study. This is the first feasibility study which investigated anti-MUC1 antibody in NAF and its relationship with tumor characteristics. Our results warrant a larger study on anti-MUC1 antibodies in NAF to validate the relationship between tumor aggressiveness and immune biomarkers.

## 5. Conclusion

This result shows that NAF containing anti-MUC1 antibody could be a reliable biomarker to predict tumor aggressiveness in BC. A larger study will be needed to confirm these data and to investigate the potential of anti-MUC1 antibodies in NAF and serum to predict disease outcome.

## Figures and Tables

**Table 1:** Pathological characteristics of breast cancer patients.

Tumor characteristics	Patient number	%
All patients	52	100
Tumor size (T)		
T ≤ 2** **cm	38	73
T > 2** **cm	14	27
Lymph node (N)		
N = 0	39	75
N = 1, 2	13	25
Estrogen receptor		
(−)	10	19
(+)	42	81
Progesterone receptor		
(−)	13	25
(+)	39	75
Her2-neu		
(−)	41	79
(+)	8	15
Missing	3	6
Triple negative		
Absent	43	83
Present	6	11
Missing	3	6
Tumor grade		
Grade 1	10	19
Grade 2	27	52
Grade 3	15	29
LVI		
Absent	42	81
Present	7	13
Missing	3	6
ECE		
Absent	48	92
Present	4	8
Ki-67 receptor		
<15	10	19
≥15	13	25
Missing	29	56

LVI: lymphovascular invasion and ECE: extracapsular extension.

**Table 2:** Anti-MUC1 antibody levels of breast cancer patients in NAF and serum samples.

Anti-MUC1 levels (mean ± SD) (OD 405 nm)						
Tumor characteristic^*∗*^	IgG	*P*	IgM	*P*	IgA	*P*
NAF (*n* = 44)						
Triple negative		**0.02**		0.12		0.90
Absent (*n* = 37)	0.10 ± 0.07		0.17 ± 0.16		0.23 ± 0.16	
Present (*n* = 6)	0.20 ± 0.20		0.29 ± 0.23		0.24 ± 0.09	
Her2-neu		0.42		0.35		**0.02**
(−) (*n* = 37)	0.11 ± 0.11		0.17 ± 0.16		0.21 ± 0.12	
(+) (*n* = 6)	0.14 ± 0.08		0.25 ± 0.25		0.36 ± 0.25	
LVI		0.85		0.75		**0.005**
(−) (*n* = 36)	0.11 ± 0.11		0.19 ± 0.19		0.21 ± 0.12	
(+) (*n* = 6)	0.10 ± 0.27		0.16 ± 0.10		0.40 ± 0.25	

Serum (*n* = 51)						
Estrogen receptor		**0.01**		0.93		0.70
(−) (*n* = 9)	0.53 ± 0.83		0.99 ± 0.48		0.26 ± 0.09	
(+) (*n* = 42)	0.16 ± 0.19		0.97 ± 0.60		0.29 ± 0.19	
Tumor size (T)				**0.03**		
T ≤ 2 cm (*n* = 38)	0.26 ± 0.46		1.08 ± 0.63		0.27 ± 0.15	
T > 2 cm (*n* = 13)	0.12 ± 0.08		0.68 ± 0.28		0.32 ± 0.25	
Recurrence		**0.01**		0.86		0.24
Absent (*n* = 47)	0.18 ± 0.21		0.98 ± 0.59		0.29 ± 0.18	
Present (*n* = 4)	0.70 ± 1.30		0.93 ± 0.59		0.18 ± 0.10	

^*∗*^One missing data in NAF sample for triple negativity and Her2-neu status and two missing data in NAF samples for LVI status from 44 malignant breast lesions. One missing data for serum samples from 52 patients with malignant. LVI: lymphovascular invasion and NAF: nipple aspirate fluid.
